# Belladonna in Spasmodic Asthma

**Published:** 1874-11

**Authors:** George C. Wood


*PHILADELPHIA MEDICAL TIMES—September*, 1874.

Belladonna in Spasmodic Asthma.—*By George C. Wood, M.D.* The communication in the *Philadelphia Medical Times* for August 29, on "Chloral Hydrate and Bromide of Potassium in Spasmodic Asthma," by Dr. Julio J. Lamadrid, has led me to offer my experience with belladonna in the same disease. Being located in a neighborhood where spasmodic asthma abounds plentifully in the autumn, I have had a fine opportunity for testing the value of the various remedies recommended for its treatment. Of all tried, which includes the hydrate of chloral and bromide of potassium, of whose use in spasmodic asthma in this country Dr. Lamadrid claims priority, I greatly prefer belladonna. It is only when belladonna, after a good trial, proves to be contraindicated, for reasons I shall hereafter state, that I make use of chloral; then I consider it the next most available remedy. Bromide of potassium has failed to produce much effect in the cases where I have tried it, either in conjunction with chloral or alone.

Belladonna, by actual experiments on animals, has been found to dilate the bronchial tubes, and keep them dilated so long as the animal remains under the influence of the drug. And, further, this dilatation persists, notwithstanding irritants be employed for the purpose of making them contract. These experiments very satifactorily account for the medicinal action of belladonna in the treatment of spasmodic asthma.

The pathology of the disease teaches us "that it is owing to a spasmodic constriction of the smaller bronchial tubes, by tonic contraction of their involuntary muscular fibres."

Belladonna, then, acts simply by relieving this constriction of the bronchial tubes.

To get the good effects of belladonna in asthma, it must be given in heroic doses. I usually employ the tincture of the United States Pharmacopœia, in doses ranging from twenty to sixty drops. The strength of the tincture differs so much, as commonly kept in the shops, that the size of the dose must be lost sight of, and the quantity given be regulated by the effect produced. It may be given during the paroxysm with great advantage, but it acts best when given before the attack commences. For example, if the patient has nocturnal attacks coming on after midnight, as is usual, give him a dose just before going to bed, and repeat it if necessary to produce sound sleep. He fails to awake at the usual time for the attack to commence, and sleeps on, awakening in the morning very much refreshed and strengthened. The treatment may be repeated night after night, until sufficient time has been had to remove the tendency of the disease to return, either by changing his location or adopting other requisite treatment, as the case may call for. I could relate several cases to prove the above statements, but will have to omit them for want of space.

Sometimes, but not often, belladonna produces dryness of the fauces, and delirium. These are indications which show that it should be discontinued and hydrate of chloral should be employed in its stead. It may be used on the same principles as belladonna to produce sleep and thus ward off attacks. For the past two years I have been treating spasmodic asthma on these principles, and with most satisfactory results; yet I do not claim any originality in their conception—they are simply hints gathered from many sources, their value being well proven, to my mind, by experience.

